# Perspective validation of the eurofever classification criteria for monogenic periodic fevers

**DOI:** 10.1186/1546-0096-12-S1-P82

**Published:** 2014-09-17

**Authors:** Silvia Federici, P Dolezalova, L Cantarini, E Papadopoulou-Alataki, M Alessio, T Herlin, I Gueli, C Modesto, G Fabio, MC Maggio, MJ Rua Elorduy, F Garibotto, A Insalaco, A Kozlova, J Anton, R Brik, J Frenkel, E Hoppenreijs, MP Sormani, Alberto Martini, Marco Gattorno

**Affiliations:** 1 UO Pediatria 2, IRCCS Gaslini, Genova, Italy; 2 Charles University in Prague and General University Hospital, Prague, Czech Republic; 3 Policlinico le Scotte, Rheumatology Unit, University of Siena, Siena, Italy; 4 Aristotle University of Thessaloniki Papageorgiou Hospital, Thessaloniki, Greece; 5 Universita' di Napoli Federico II, Napoli, Italy; 6 Skejby Sygehus, Aarhus University Hospital, Aarhus, Denmark; 7 Hospital Valle de Hebron, Barcelona, Spain; 8 Fondazione IRCCS Ca' Granda , Ospedale Maggiore Policlinico, Milano, Spain; 9 Ospedale dei Bambini, Palermo, Italy; 10 Hospital de Cruces, Bilbao Vizcaya, Spain; 11 Ospedale Pediatrico Bambin Gesu', Rome, Italy; 12 Federal Research Center of Pediatric Hematology, Oncology and Immunology, Moscow, Russian Federation; 13 Hospital Sant Joan de Déu, Universitat de Barcelona, Barcelona, Spain; 14 Rambam Medical Center, Haifa, Israel; 15 Department of Paediatrics, University Medical Center Utrecht, Utrecht; 16 UMC St Radboud Ziekenhuis, Nijmegen, Netherlands

## Introduction

We recently proposed a set of provisional, evidence-based, clinical criteria for the classification of children and adults patients affected by monogenic periodic fevers. These criteria, built and validated in a cohort of 1215 patients with periodic fever enrolled in the Eurofever Registry from November 2009 to February 2013, displayed a high sensitivity and specificity.

## Objectives

To prospectively validate the provisional Eurofever clinical classification criteria in an independent set of patients with periodic fevers enrolled in the registry after March 2013

## Methods

The provisional Eurofever classification criteria (best accuracy cut-off) were applied to patients with periodic fevers enrolled in the registry after March 2013. The following disease were analyzed: FMF, MKD, TRAPS, CAPS, PFAPA and undefined periodic fevers. Patients were divided in three subgroups: i) group A: inherited periodic fevers with genetic confirmation, ii) group B: inherited periodic fevers with non-confirmatory genetic test, iii) group C: negative controls. Statistical analysis compared the sensitivity and specificity of best accuracy cut-off in patients belonging to group A and C.

## Results

A total of 245 patients were evaluated. Among them 80 were classified in group A (35 FMF, 28 MKD, 9 TRAPS, 8 CAPS), 94 in group B (56 FMF, 6 MKD, 9 TRAPS, 23 CAPS) and 71 in group C (59 PFAPA and 12 undefined periodic fever). The classification criteria tested on patients belonging to Group A and C displayed an overall good sensitivity and specificity (respectively 85% and 92% for FMF, 92% and 89% for MKD, 100% and 96% for CAPS and 100% and 93% for TRAPS). (see table 1). The performance of the classification criteria in patients with non- confirmatory genetic test are reported in Table 1 below.

**Table 1:** 

	Sensitivity (Best Accuracy cut-off)				Specificity (Best Accuracy cut-off)			
	**FMF**	**MKD**	**CAPS**	**TRAPS**	**FMF**	**MKD**	**CAPS**	**TRAPS**

Genetically confirmed (Group A)	85%	92%	100%	100%	92%	89%	96%	93%

Not genetically confirmed (Group B)	58%	67%	45%	25%	94%	79%	100%	82%

## Conclusion

Perspective validation of Eurofever classification criteria showed their good accuracy in patients with confirmatory genetic test. Despite a lower sensitivity, the criteria displayed an high specificity in the classification of patients with non-confirmatory molecular test. In order to evaluate the actual accuracy of the classification criteria in this latter subgroup, an expert validation is currently ongoing.

## Disclosure of interest

None declared.

